# Clinical Survey and Predictors of Outcomes of Pediatric Out-of-Hospital Cardiac Arrest Admitted to the Emergency Department

**DOI:** 10.1038/s41598-019-43020-0

**Published:** 2019-05-07

**Authors:** Jung Lee, Wen-Chieh Yang, En-Pei Lee, Jing-Long Huang, Hsiang-Ju Hsiao, Mao-Jen Lin, Han-Ping Wu

**Affiliations:** 1 Division of Pediatric General Medicine, Department of Pediatrics, Chang Gung Memorial Hospital at Linko, Kweishan, Taoyuan, Taiwan; 2 College of Medicine, Chang Gung University, Taoyuan, Taiwan; 3 Department of Pediatric Emergency Medicine, Children Hospital, China Medical University, Taichung, Taiwan; 4 Department of Medicine, School of Medicine, China Medical University, Taichung, Taiwan; 5 Division of Pediatric Critical Care Medicine, Department of Pediatrics, Chang Gung Memorial Hospital at Linko, Kweishan, Taoyuan, Taiwan; 6 Division of Pediatric Allergy, Asthma, and Rheumatology, Department of Pediatrics, Chang Gung Memorial Hospital at Linko, Kweishan, Taoyuan, Taiwan; 7 Division of Cardiology, Department of Medicine, Taichung Tzu Chi Hospital, The Buddhist Tzu Chi Medical foundation, Taichung, Taiwan; 8 Department of Medicine, School of Medicine, Tzu Chi University, Hualien, Taiwan; 9 Department of Medical Research, Children’s Hospital, China Medical University, Taichung, Taiwan

**Keywords:** Risk factors, Paediatric research

## Abstract

Pediatric out-of-hospital cardiac arrest (OHCA) is a rare event with severe sequelae. Although the survival to hospital-discharge (STHD) rate has improved from 2–6% to 17.6–40.2%, only 1–4% of OHCA survivors have a good neurological outcome. This study investigated the characteristics of case management before and after admittance to the emergency department (ED) associated with outcomes of pediatric OHCA in an ED. This was a retrospective study of data collected from our ED resuscitation room logbooks dating from 2005 to 2016. All records of children under 18 years old with OHCA were reviewed. Outcomes of interest included sustained return of spontaneous circulation (SROSC), STHD, and neurological outcomes. From the 12-year study period, 152 patients were included. Pediatric OHCA commonly affects males (55.3%, n = 84) and infants younger than 1 year of age (47.4%, n = 72) at home (76.3%, n = 116). Most triggers of pediatric OHCA were respiratory in nature (53.2%, n = 81). Sudden infant death syndrome (SIDS) (29.6%, n = 45), unknown medical causes (25%, n = 38), and trauma (10.5%, n = 16) were the main causes of pediatric OHCA. Sixty-two initial cardiac rhythms at the scene were obtained, most of which were asystole and pulseless electrical activity (PEA) (93.5%, n/all: 58/62). Upon ED arrival, cardiopulmonary resuscitation (CPR) was continued for 32.66 ± 20.71 min in the ED and 34.9% (n = 53) gained SROSC. Among them, 13.8% (n = 21) achieved STHD and 4.6% (n = 7) had a favorable neurological outcome. In multivariate analyses, fewer ED epinephrine doses (*p* < 0.05), witness of OHCA (*p* = 0.001), and shorter ED CPR duration (*p* = 0.007) were factors that increased the rate of SROSC at the ED. A longer emergency medical service (EMS) scene interval (*p* = 0.047) and shorter ED CPR interval (*p* = 0.047) improved STHD.

## Introduction

Pediatric out-of-hospital cardiac arrest (OHCA) is a rare event associated with poor outcomes.1 The incidence of OHCA varies among countries, ranging from 2.28 to 18.0/100,000 person-years^1–12^. Previously, the survival to hospital discharge (STHD) rate of pediatric OHCA was 2–6%^4,5,13–15^ and with the advance of pediatric emergency medicine, this has improved to 17.6–40.2%^16–18^. For those who survive, only 1–4% of them have good neurological outcomes^4,5,13–15^. Poor outcomes of pediatric OHCA have been related to patient, cardiac event, resuscitation, and post-resuscitation care factors^19^. Managing pediatric OHCA efficiently is a vital challenge for physicians in the emergency department (ED). Information on factors associated with post-OHCA prognosis can facilitate improvement in pre- and in-ED care; improving survival with good neurological outcomes^20^. Identification and documentation of aspects other than epidemiological variables of pediatric OHCA are of great importance for developing a treatment plan and determining proper preventive measures. This study assessed the clinical characteristics, prior to and during admission to the ED, associated with clinical outcomes including sustained return of spontaneous circulation (SROSC), STHD, and neurological outcomes of pediatric OHCA in an ED.

## Methods

### Study setting and patient selection

This study was conducted in the ED of Chang Gung Hospital in Taiwan. The setting of our study was a tertiary medical center that receives cases transferred from local clinics and regional hospitals. The data were collected from the ED resuscitation room logbooks from January 2005 to December 2016. All records of children under 18 years old who were pulseless on arrival and required cardiac pulmonary resuscitation (CPR) at the ED were reviewed. The study was approved by the Institutional Review Board of the Chang Gung Memorial Hospital (201701095B0). All methods were performed in accordance with the relevant guidelines and regulations.

The data were collected, reviewed, de-identified, and anonymously analyzed by the authors, and the ethics committee waived the requirement for informed consent because of the anonymized nature of the data and scientific purpose of the study.

### Study design

Patients who were of gestational age of less than 21 weeks, had “do not resuscitate” orders, or were transferred from another ED after return of spontaneous circulation (ROSC) were excluded. Data collected included patient demographic profiles (i.e., age, sex), category of etiology, comorbidities, timing, initial heart rhythm, place, events, and history that involved the OHCA. Pre-ED information was obtained from the emergency medical services (EMS) records, including the time the call was received, the time of arrival and departure from the scene, the time the patient arrived at the ED, and the duration of pre-ED CPR. Places of OHCA were classified as home/residence, industrial/workplace, sports/recreation event, street/highway, public building, daycare/nursing home, educational institution, other, and unknown/not documented. Duration of transportation, timing of interventions by a bystander, EMS, and physicians were also recorded and analyzed. Response interval was defined as the time from incoming call to the time the first emergency response vehicle stopped at a point close to the patient’s location^21^. The duration of ED CPR was defined as the time interval from the time CPR was initiated to the time it was stopped at the ED. Outcomes including SROSC, STHD, and pediatric cerebral performance category (PCPC) score were collected and analyzed^21^. SROSC was defined as restoration of perfusing and heart rhythm in the absence of external chest compressions for over 20 min^14^. The PCPC scores, ranging from 1 (normal) to 6 (brain dead), were validated to quantify a child’s cognitive function after a critical illness or an injury. The investigators judged the PCPC score by reviewing the discharge summaries and outpatient records with the consensus of another investigator. Categories 1 to 3 were viewed as good neurological outcomes^22^. The American Heart Association (AHA) guidelines for CPR was updated to the chest compressions, airway, breathing/ventilations (CAB) sequence from the conventional airway, breathing/ventilations, chest compressions (ABC) sequence in 2010^23^. The trend of outcomes before and after this change was evaluated for a 5-year time span.

### Statistical methods

Descriptive statistics are presented for most variables (i.e., demographics). Univariate summaries (means, standard deviations) were provided for continuous variables (e.g., age), whereas frequencies and percentages were used to summarize categorical variables (e.g., sex). SPSS ver. 21 software was used for all analyses and a p-value < 0.05 was considered to reflect statistical significance. Student t test and the χ^2^ test with the Fisher exact test were used to test the significance of categorical and numerical variables, respectively. Multivariate logistic regression analyses were performed to determine the factors in regard to outcomes of pediatric OHCA. Variables were kept in the final model if the p value was <0.05.

## Results

### Demographics

We retrieved 12 years of records from 2005 to 2016, featuring 217 OHCA cases. Children transferred from other EDs after ROSC (n = 59), who were extremely premature with a gestational age of less than 21 weeks (n = 2), and those with “do not resuscitate” orders (n = 4) were excluded. The 217 OHCA cases represented 23 of every 100,000 pediatric ED visits of a total 946,877 pediatric ED visits in 12 years. Of the final 152 OHCA cases, 55.3% (n = 84) were male. Of all cases, 47.4% (n = 72) were aged less than 1 year old (Y/O), 28.3% (n = 43) were 1–6 Y/O, 10.5% (n = 16) were 7–12 Y/O, and 13.8% (n = 21) were 13–18 Y/O (Table 1).

**Table 1:** Child characteristics and associations with outcomes.

	All	%	SROSC		*p*	STHD		*p*	PCPC ≤ 3	
			Yes	No		Yes	No		Yes	No
	N = 152		N = 53	N = 99		N = 21	N = 131		N = 7	N = 14
**Demographic and prevention**										
Age (y/o)										
<1(n/all)	72/152	47.4	30/53	42/99		14/21	58/131		7/7	7/14
1~6(n/all)	43/152	28.3	13/53	30/99		2/21	41/131		0	2/14
7~12(n/all)	21/152	13.8	5/53	11/99		2/21	19/131		0	2/14
13~18(n/all)	16/152	10.5	5/53	16/99	0.37	3/21	13/131	0.17	0	3/14
Gender										
Male (n/all)	84/152	55.3	32/53	52/99	0.39	10/21	74/131		3/7	7/14
Female (n/all)	68/152	44.7	21/53	47/99	0.39	11/21	57/131	0.48	4/7	7/14
Pre-existing condition										
Yes (n/all)	56/152	36.8	23/53	33/99		10/21	46/131		3/7	7/14
No (n/all)	96/152	63.2	30/53	66/99	0.29	11/21	85/131	0.33	4/7	7/14
**Early cardiopulmonary resuscitation (CPR)**										
Witnessed of OHCA										
Yes (n/all)	64/152	42.1	36/53	28/99		17/21	47/131		7/7	10/14
No (n/all)	88/152	57.9	17/53	71/99	<*0*.*05*	4/21	84/131	<*0*.*05*	0/7	4/14
Bystander CPR										
Yes (n/all)	15/152	9.9	9/53	6/99		1/21	14/131		1/7	0
No (n/all)	137/152	90.1	44/53	93/99	*0*.*045*	20/21	117/131	0.69	6/7	14/14
**Prompt access to the emergency response system**										
Sent by EMS (n/all)	80/152	52.6	25/53	55/99		10/21	70/131		3/7	7/14
Sent by caretaker (n/all)	72/152	47.4	28/53	44/99	0.39	11/21	61/131	0.64	4/7	7/14
EMS advanced airway	n = 62		n = 16	n = 46		n = 8	n = 54			
Yes (n/all)	12	19.4	2/16	10/46		2/8	10/54			
No (n/all)	50	80.6	14/16	32/46	0.71	6/8	44/54	0.64		
**Rapid pediatric advanced life support (PALS)**										
Pediatrician										
Yes (n/all)	107/152	70.4	43/53	64/99		19/21	88/131		7/7	12/14
No (n/all)	45/152	20.6	10/53	35/99	*0*.*041*	2/21	43/131	<*0*.*05*	0	2/14
Shift										
Day shift (n/all) (7 A.M. to 7 P.M.)	89/152	58.6	33/53	56/99		14/21	75/131		5/7	9/14
Night shift (7 P.M. to 7 A.M) (n/all)	63/152	41.4	20/53	43/99	0.6	7/21	56/131	0.48	2/7	5/14
ED CPR	Mean ± SD		Mean ± SD	Mean ± SD		Mean ± SD	Mean ± SD			
ED Epinephrine doses	8.79 ± 6.21 (n = 151)		3.85 ± 3.68 (n = 52)	11.38 ± 5.68 (n = 99)	<*0*.*05*	2.14 ± 2.81 (n = 21)	9.86 ± 5.94 (n = 130)	<*0*.*05*		
First epinephrine time (min) at ED	2.92 ± 3.12 (n = 142)		3 ± 3.25 (n = 43)	2.9 ± 3.08 (n = 99)	0.86	2.64 ± 3.91 (n = 14)	2.96 ± 3.04 (n = 128)	0.71		
ED CPR interval (min)	32.66 ± 20.71 (n = 152)		17.3 ± 16.39 (n = 53)	40.89 ± 17.96 (n = 99)	<*0*.*05*	9.76 ± 9.57 (n = 21)	36.34 ± 19.65 (n = 131)	<*0*.*05*		

Minutes (min).

### Prevention

The home/residence was the most common location of OHCA (76.3%, n = 116), followed by the street/highway (10.5%, n = 16), daycare location/nursing home (7.2%, n = 11), educational institution (2%, n = 3), unknown (2%, n = 3), sports/recreation venue (1.3%, n = 2), and public building (0.7%, n = 1). Pediatric OHCA occurred often in the morning (n = 56, 36.8%), in January and March (34.3%, n = 52). Ninety-six patients (63.2%) were well while 56 children (36.8%) had preexisting conditions: 13.8% (n = 21) neurological conditions/seizures, 8.6% (n = 13) prenatal complications, 6.6% (n = 10) cardiac conditions, 2.6% (n = 4) congenital anomalies, 2% (n = 3) failure to thrive, 1.3% (n = 2) cancer, 1.3% gastrointestinal problems (n = 2), and 0.7% (n = 1) systemic lupus erythematous.

The causes of pediatric OHCA are listed in Table 2. Sudden infant death syndrome (SIDS) (29.6%, n = 45), unknown medical cause (25%, n = 38), and trauma (10.5%, n = 16) were the top three causes. SIDS was mostly observed in infants (62.5%, n/all: 45/72) and trauma was most common in those 13–18 Y/O (62.5%, n/all: 10/16). The initial physiological compromise was respiratory in nature for 81 patients (53.2%)

**Table 2:** Manner of pediatric OHCA.

Manner of pediatric OHCA	Frequency
Sudden infant death syndrome (SIDS)	45 (29.6%)
Unknown medical causes	38 (25%)
Trauma (Traffic accident (n = 9), 5 Fall (n = 5), Other injury (n = 2)	16 (10.5%)
Choking	15 (9.8%)
Suspected child abuse	11 (7.2%)
Precipitate delivery	8 (5.3%)
Drowning	5 (3.3%)
Epilepsy	4 (2.6%)
House fire	3 (2%)
Ventilator out	2 (1.3%)
Suicide	1 (0.7%)
CO intoxication	1 (0.7%)
Hanged	1 (0.7%)
Myocarditis	1 (0.7%)
Drug overdose (amphetamine)	1 (0.7%)
Total	152

CO: carbon monoxide.

### Early cardiopulmonary resuscitation

OHCA was witnessed in 64 (42.1%) children, and bystander CPR was delivered in 15 (9.9%) cases, including conventional CPR (5.9%, n = 9) and chest compression-only CPR (4.0%, n = 6), but no ROSC was recorded at scene. Witness of an OHCA was associated with SROSC (*p* < 0.05) and STHD (*p* < 0.05). Bystander CPR was associated with SROSC (*p* = 0.045), but not STHD (*p* = 0.69).

### Prompt access to the EMS

Eighty (52.6%) cases were transferred by EMS and 62 records were available. All underwent life support with oxygenation and chest compressions. Neither medications nor fluids were administrated by EMS due to lack of intravenous (IV) access. Bag-valve-mask (BVM) ventilation (77.4%, n = 48), laryngeal mask airway (LMA) assistance (19.4%, n = 12), and non-rebreathing masks (3.2%, n = 2) were used for oxygenation. Pre-ED advanced airway management (19.4%, n = 12) was associated with neither SORSC (p = 0.71) nor STHD (p = 0.64) (Table 1). EMS records (Table 3) showed that the total transport interval was 21.16 ± 12.96 min including the response interval of 6.52 ± 6.7 min, the scene interval of 6.53 ± 4.6 min, and the scene to ED interval of 8.27 ± 7.42 min. Fifty-nine (95.1%) cases reported response intervals of <10 min. All patients received EMS CPR for 11.61 ± 7.07 min. The interval from EMS arrival to the time of the first epinephrine exposure was 17.28 ± 8.7 min (mean ± SD) in 61 records and 96.7% (n = 59) had their first epinephrine exposure after over 10 min. No EMS event intervals were significant for outcomes. A longer scene interval was associated with STHD (11.0 ± 6.5 vs. 5.85 ± 3.92 min, *p* = 0.02) but not SROSC (7.25 ± 6.12 vs. 6.26 ± 4.0 min, *p* = 0.46).

**Table 3:** Time frame of emergency response system (min).

EMS	All	SROSC		*P* value	STHD		*p* value
	Mean ± SD (n = 62)	Mean ± SD (n = 16)	Mean ± SD (n = 46)		Mean ± SD (n = 8)	Mean ± SD (n = 54)	
EMS response interval (min)	6.45 ± 6.70	5.88 ± 3.64	6.65 ± 7.50	0.69	5.25 ± 2.25	6.63 ± 7.13	0.59
EMS scene interval (min)	6.53 ± 4.60	7.25 ± 6.12	6.26 ± 4.00	0.46	11.0 ± 6.5	5.85 ± 3.92	*0*.*02*
EMS Scene to ED interval (min)	8.27 ± 7.41	7.69 ± 4.19	8.48 ± 8.27	0.71	8.25 ± 5.41	8.28 ± 7.70	0.99
EMS total transport interval (min)	21.16 ± 12.96	20.5 ± 7.09	21.39 ± 14.51	0.81	24.5 ± 4.72	20.67 ± 13.72	0.44
EMS CPR interval (min)	11.61 ± 7.07	11.94 ± 7.12	11.49 ± 7.14	0.83	14.38 ± 7.68	11.19 ± 6.96	0.23
	All	SROSC			STHD		
EMS arrival to first epinephrine interval (min)	17.28 ± 8.7 (n = 61)	17.2 ± 5.89 (n = 15)	17.30 ± 9.58 (n = 46)	0.96	21.57 ± 5.06 (n = 7)	16.72 ± 9.03 (n = 54)	0.17

Initial cardiac rhythms at the scene were asystole/pulseless electrical activity (PEA) (93.5%, n/all: 58/62), ventricular tachycardia/fibrillation (VT/VF) (4.84%, n/all: 3/62), and bradycardia (1.6%, n/all: 1/62). Three VT/VF cases underwent defibrillation via EMS-administered automated external defibrillation (AED); two exhibited ROSC during transport but arrested in the ED.

### Rapid pediatric advanced life support (PALS)

All patients were ventilated via endotracheal intubation (89.5%, n = 136), BVM (8.6%, n = 13), or tracheostomy (1.9%, n = 3) in the ED. ED medication records were available for 151 patients, and these showed that 142 (94%) patients received epinephrine for 8.79 ± 6.21 doses during CPR and received their first epinephrine exposure at 2.92 ± 3.12 min from ED arrival. For the route of epinephrine administration, 127 (89.4%) were treated via IV, 6 (4.2%) by endotracheal tube, and 9 (6.3%) by endotracheal tube followed by IV. Fewer doses of epinephrine in ED CPR were associated with SROSC (p < 0.05) and STHD (p < 0.05) but the first epinephrine exposure time in the ED was neither associated with SROSC (3 ± 3.25 vs. 2.9 ± 3.08 min, p = 0.86) nor STHD (2.64 ± 3.91 vs. 2.96 ± 3.04 min, p = 0.71). The average ED CPR interval was 32.66 ± 20.71 min. A shorter ED CPR interval was associated with both SROSC (17.3 ± 16.39 vs. 40.89 ± 17.96 min, *p* < 0.05), and STHD (9.76 ± 9.57 vs. 36.34 ± 19.65 min, *p* < 0.05) (Table 1). The initial heart rhythms at ED triage were asystole (96.7%, n = 147), VT/VF (1.3%, n = 2), bradycardia (1.3%, n = 2), and PEA (0.7%, n = 1). Two cases with initial VT/VF were shocked and one achieved STHD. The shockable rhythms during CPR were VT/VF in 13 (8.6%) cases; all were defibrillated. In total, 107 cases (70.3%) were resuscitated by pediatricians. Compared to non-pediatricians, pediatricians attained a higher SROSC rate (*p* = 0.041) and STHD rate (*p* < 0.05).

### Post-cardiac arrest care

**Figure 1:**
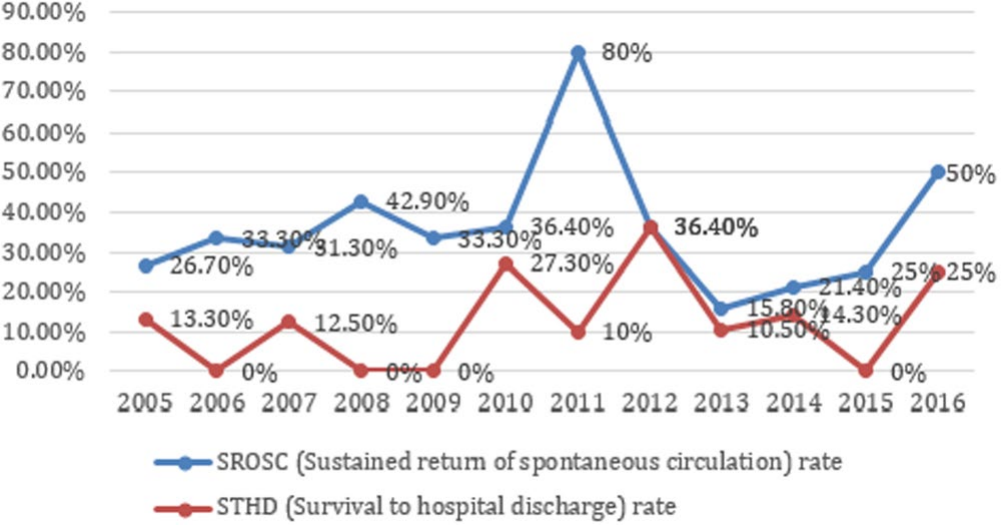
The trend of outcomes for all 152 patients over time before and after PALS update in 2010.

**Figure 2:**
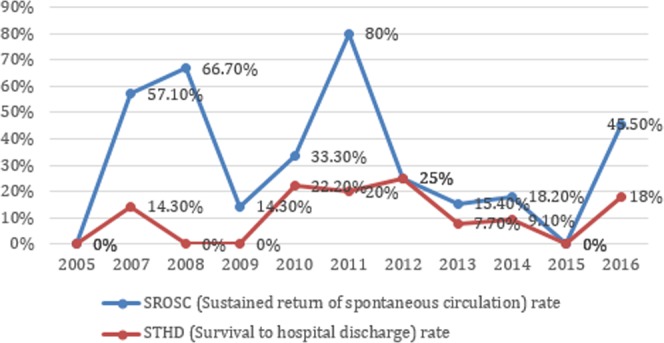
The trend of outcomes for 80 patients who receive emergency medical service (EMS) transfer over time before and after PALS update in 2010.

Overall, 34.9% (n = 53) of cases developed SROSC, 13.8% (n = 21) STHD, and 4.6% (n = 7) had favorable neurological outcomes (PCPC scores 1–3) (Table 1). Sixteen (10.5%) patients survived less then 24 h and the 1-day, 7-day, 30-day, 90-day, and 180-day survival rates were 24.3% (n = 37), 17.7% (n = 27), 13.8% (n = 21), 11.2% (n = 17), and 9.86% (n = 15). The trend of outcomes over time before and after the PALS update in 2010 was plotted (Figs 1 and 2). For the 99 cases of failed CPR, nearly half (44.4%) had no postmortem exam for evaluation.

### Factors of outcomes

After univariate analyses, significant variables for SROSC included witness of OHCA (*p* < 0.05), bystander CPR (*p* = 0.045), pediatrician (*p* = 0.041), ED epinephrine doses (*p* < 0.05), and ED CPR interval (*p* < 0.05) were entered into the logistic regression model. In multivariate analyses, fewer ED epinephrine doses (*p* < 0.05), witness of OHCA (*p* = 0.001), and shorter ED CPR duration (*p* = 0.007) were significant for SROSC (Table 4). For STHD, witness of OHCA (*p* < 0.05), pediatrician (*p* = 0.041), ED epinephrine doses (*p* < 0.05), ED CPR interval (*p* < 0.05), and EMS scene interval (*p* = 0.02) were entered into multivariate analyses and a longer EMS scene interval (p = 0.047) and shorter ED CPR interval (p = 0.047) were kept in the model (Table 5). Age, sex, preexisting condition, day or night OHCA, EMS transport, and EMS advanced airway management were not associated with the outcomes of interest. We could not determine predictors of neurological outcomes because of the small number of cases.

**Table 4:** Multivariate analysis for factors associated with SROSC.

Parameters	Adjusted Odds ratio	*P* value
Witness of OHCA	0.141	0.001*
Bystander CPR	0.153	0.058
Pediatrician	0.845	0.78
ED epinephrine doses	0.724	<0.05*
ED CPR interval	0.951	0.007*

*Statistical significance was set at *p* < 0.05.

**Table 5:** Multivariate analysis for factors associated with STHD.

Parameters	Adjusted Odds ratio	*P* value
Witness of OHCA	0.177	0.311
EMS scene time	1.56	0.047*
Pediatrician	0.386	0.605
ED epinephrine doses	0.947	0.769
ED CPR interval	0.799	0.047*

*Statistical significance was set at *p* < 0.05.

## Discussion

Pediatric OHCA cases represented 23 of every 100,000 pediatric visits in our ED. The most common age was infancy^5,14,18^ and the incidence was higher in males^1,5,13^. Medically fragile children (with preexisting conditions; 30.6%) were also at a high risk for OHCA^13^. The overall SROSC rate was 34.9%, higher than in an earlier report (a pooled study rate of 27.8%)^14^. The STHD rate was 13.8%, within the range of earlier studies (4.7–40.2%)^5,14,16–18,24–26^. Of our patients, 4.6% had a good neurological outcome which matched our earlier figure of 1–4%^4,5,13–15^.

The common causes of pediatric OHCA were consistent with previous reports: SIDS, airway-related causes, trauma, drowning, intoxication, and cardiac causes^5,10,27^ most triggers were respiratory in nature^28,29^. The home/residence was the most common site of OHCA (76.3%, n = 116) (60% in a previous report)^4^ and the rate was higher between 00:00 and 09:0029 in Spring. SIDS was the principal cause of pediatric OHCA; the rate was similar to those of other studies (18–60%)^2,10,13,25,26,30,31^. The high prevalence of SIDS at home in the early morning must be recognized when seeking to prevent and monitor this phenomenon. Unknown medical causes were the second common OHCA trigger, rendering precise etiological diagnosis difficult. Notably, 63.2% of children had no preexisting condition and 44.4% did not undergo blood testing or imaging, indicating that OHCA etiologies were underexplored. If the clinical cause of OHCA is not that of the coroner’s report^11^, autopsy might clarify the exact cause of death. Potentially preventable etiologies including trauma, suspected abuse, choking, drowning, poor ventilation, house fires, and hanging accounted for 35.5% of all OHCAs. Child death review seeking to improve strategies for preventing childhood deaths caused by preventable etiologies is imperative^32^.

The OHCA witness rate was earlier reported to be 30.8–34%, thus lower than in our study (42.1%)^1,13,14,18^. Witnessed arrest status was significantly associated with SROSC and STHD, as also reported in an earlier pooled study^14^, due to immediate emergency response when witnessing reduced the no-blood-flow time^1,14,33^. Although our witness rate was higher than those of other reports, the bystander CPR rate (9.9%) was lower than a previous report of 17–35%^1,2,5,10,13,25,26,30^. Our bystander CPR frequency was low even when OHCA was witnessed^13^, indicating that public resources on pediatric life support were insufficient and caretakers were unfamiliar with pediatric life support^13^. This emphasizes the need to the improve bystander CPR rate^5^ through education or reachable instructions of pediatric life support, particularly for potential caregivers of medically high-risk children^13^.

Our results are consistent with the notion that bystander CPR is associated with SROSC but not STHD^14^. It cannot be assumed that bystander CPR will aid survival because the no-blood-flow time prior to CPR may be considerable, and many post-SROSC factors may affect STHD.

Nearly half (52.6%) of patients were transported by EMS, a much lower rate than that reported in previous study (81%)^5^. Our OHCA cases transported by EMS were all supported with oxygenation and chest compressions while en route to the ED. Surprisingly, the outcomes of those patients did not differ from those of children transported by caregivers, which suggests that pre-ED oxygenation and chest compression may be insufficient to improve outcomes of pediatric OHCA. The primary deficiency of EMS service in this study was a lack of or failure to procure IV access for epinephrine and fluid administration. Early epinephrine administration was associated with better survival and favorable neurological outcomes^34^ and intraosseous administration is a safe and effective method for delivering drugs during CPR^35^.

EMS systems should consider strategies such as the use of IO for early epinephrine administration in pediatric OHCA whenever IV access cannot be rapidly obtained^34^. Our rate of advanced airway management (19.4%) was higher than that in a previous study (16.9%)^36^, but pre-ED EMS advanced airway management failed to increase both SRSC and STHD^36^. The most common presenting arrhythmia at the scene was asystole (68–92%)^5,13,14,37^ because most arrests were due to asphyxiation and were unwitnessed, triggering progressive hypoxia and ultimately cardiac arrest with asystole^28^. Timely defibrillation of shockable rhythms (VT and VF) is critical in terms of survival^38^. Unfortunately, the rate of VT/VF at the scene was lower than that of previous pediatric studies (2–19%)^5,13,14,37^. Placement of defibrillators in public places and training of volunteers would promote earlier identification and defibrillation, and thus improve survival^39^. A longer EMS scene interval, which implies more resuscitation efforts at the scene rather than a “scoop and run” approach, was associated with STHD in this study^5^. This indicates that EMS resuscitation approaches for children at the scene are important and differences in EMS practice at the scene deserve more research to improve outcomes^5^.

Pediatricians achieved higher SROSC and STHD rates than non-pediatricians in univariate analyses. Michelson *et al*. also reported higher pediatric survival after non-traumatic OHCA when patients were in pediatric EDs rather than general EDs^40^. Although this item did not remain in the model of multivariate analyses, pediatricians whose resuscitation practice is constantly refreshed in real pediatric environments should improve the outcomes of pediatric OHCA and all emergency care providers should seek more training in real or simulated child resuscitation.

High-fidelity simulation (HFS), which replace real-life experiences with guided experience in realistic clinical situations, are routinely used to train professionals to cope with high-risk and/or lower-frequency pediatric events^41–45^. Deliberate and repetitive pediatric HFS that enhance skills, team spirit, and leadership for ED physicians will be helpful for the outcomes of pediatric OHCA^44,46^.

Fewer epinephrine doses, which augment coronary blood flow efficiently^47^, is associated with increased hospital survival for pediatric OHCA patients^27,48^. As the ED CPR duration decreased, the probabilities of SROSC and STHD increased. A short ED resuscitation time reduced the no-blood-flow time prior to SROSC, and improved outcomes. Thus, prolonged resuscitative efforts do not afford good neurological outcomes to survivors^13,26^.

In 2010, the PALS guideline substituted CPR ABC with CAB, and emphasized the importance of chest-compression-only CPR^23^. We found that the trend of SROSC and STHD rates were not significantly different. Most child OHCA is triggered by respiratory causes, not cardiac caused as in adult OHCA^28^. It is important to apply both chest compressions and rescue breathing during pediatric OHCA resuscitation^28^. Ideally, traditional CPR is recommended for pediatric OHCA, but compression-only CPR is better than no CPR^49^ The optimal CPR for pediatric OHCA should be studied further.

Both the causes and management of pediatric OHCA differ from those of adult OHCA. In adults, predictors of improved survival are the presence of an initial cardiac rhythm (ventricular fibrillation/ventricular tachycardia), bystander CPR, the time taken to travel from the scene to hospital, and early defibrillation^50–56^. In children, our pre- and in-ED data revealed several predictors of outcomes after pediatric OHCA. Fewer ED epinephrine doses^27,48^ witness of OHCA^14^ and shorter ED CPR time increased the SROSC rate. A longer EMS scene interval5 and shorter ED CPR interval^27,48^ improved STHD.

### Limitations

This was a retrospective study and the data or timing might be not well documented in a rush for OHCA events at scene or ED such as duration of out of hospital CPR, total pulseless time prior to the ED, risk-adjustment including multiple birth, gestational age, patient healthy condition, socioeconomic status, family situation for confounding bias. Those factors may cause recording bias and poor quality of data. Further studies prospectively collecting data on pediatric OHCA would be required.

## Conclusion

Pediatric OHCA affects mainly male infants younger than 1 year of age at home, with typically bad outcomes. Most triggers of pediatric OHCA are respiratory in nature and the most common arrhythmia is asystole. A witness of OHCA, shorter ED CPR interval, and fewer ED epinephrine doses increased the SROSC rate in our ED, while a longer EMS scene interval and shorter ED CPR interval improved STHD. Our work provides information aimed to improve pre- and in-ED preparations and provides public health authorities with tools for the management and prevention of pediatric OHCA.

### Ethics approval and consent to participate

The study protocol was approved by the Institution Review Board and ethics committee of Chang-Gung Memorial hospital(201701095B0).
